# An Examination of Device Types and Features Used by Adult Electronic Nicotine Delivery System (ENDS) Users in the PATH Study, 2015–2016

**DOI:** 10.3390/ijerph16132329

**Published:** 2019-07-02

**Authors:** Blair Coleman, Joanne T. Chang, Brian L. Rostron, Sarah E. Johnson, Babita Das, Arseima Y. Del Valle-Pinero

**Affiliations:** Office of Science, Center for Tobacco Products, U.S. Food and Drug Administration, Beltsville, MD 20705, USA

**Keywords:** electronic nicotine delivery systems (ENDS), e-cigarettes, population studies

## Abstract

To date no study has reported U.S. nationally representative estimates of current ENDS users by device category (“open” vs. “closed” systems) nor their detailed use behaviors. We examined the proportion of current adult ENDS users (unweighted *n* = 2671) using either “closed” or “open” systems during Wave 3 (2015–2016) of the Population Assessment of Tobacco and Health (PATH) Study. Demographic characteristics, use patterns, and device characteristics were examined overall and by device type. Among the 5.0% of current users at Wave 3, 43.9% used closed systems and 53.7% used open systems. Compared to closed system users, open system users were more likely to be male (60.7% vs. 48.4%), aged 18–24 (30.4% vs. 21.4%), and non-Hispanic White (76.2% vs. 65.4%), recent former (9.9% vs. 5.6%) or long-term former (20.2% vs. 10.9%) smokers, and use ENDS daily (44.1% vs. 22.5%); they were less likely to be to be current daily smokers (31.7% vs. 48.0%) or never smokers (15.2% vs. 19.5%). Adult ENDS users were nearly evenly split on their use of closed versus open systems; however, several group differences were observed. Disentangling the relationship between device selection and subsequent use patterns remains a public health priority.

## 1. Introduction

Prior research on electronic nicotine delivery systems (ENDS) has shown that device features—namely, device size and, related, battery strength—affect device performance and user experience [1,2,3,4]. Similarly, extant evidence suggests that device features or types are associated with different use patterns (e.g., frequency) [5,6]. As such, a better understanding of characteristics of users of different device types, and use patterns associated with device type, can provide insight on this diverse product class, including their potential impact on population health.

While a number of studies have explored device features used by adult ENDS users, [6,7,8,9] no study to date has reported U.S. nationally representative estimates of current users by device category (i.e., “open” systems [refillable with e-liquid, and components may be modifiable] versus “closed” systems [pre-filled with liquid or use pre-filled cartridges]) nor their detailed use behaviors. In this study, we provide the most up-to-date population-level estimates of users by device category (open vs. closed systems) and characterize patterns of use by these two groups, including use frequency, duration of use, dependence, use of flavors/nicotine, and device modifications.

## 2. Materials and Methods

Data are from Wave 3 of the PATH Study collected from October 2015 to October 2016. The PATH Study is an ongoing, nationally-representative longitudinal cohort study of U.S. civilian, non-institutionalized adults and youth ages ≥ 12 years. Wave 3 collected data from 28,148 adults (weighted response rate: 78.4%). Details regarding the PATH Study design and methods are published elsewhere [10].

Among adults who reported current (every day or some day) use of “electronic nicotine products” (ENPs), the PATH Study questionnaire term used to describe ENDS, primary ENP device type was assessed with a series of binary questions on device attributes. Based on prior research [6,11] device types were categorized as either “closed systems” (not rechargeable; or rechargeable and used cartridges) or “open systems” (rechargeable, did not use cartridges, and refillable). A small number of adult ENP users (*n* = 72) were dropped from device type stratification due to missing data on device attributes (*n* = 30) or incompatible combinations of responses that precluded categorization (*n* = 42).

In 2018, descriptive statistics of current ENP users, including demographic characteristics, other tobacco use, and patterns of use were examined overall and by primary device type (closed versus open systems). Analyses were conducted using SAS version 9.4 (SAS Institute, Cary, NC) using replicate weights and balanced repeated replication methods with Fay’s adjustment of 0.3 to account for the PATH Study’s complex survey design. Differences in characteristics between groups were evaluated using Rao-Scott chi-squared tests.

## 3. Results

The prevalence of current ENP use in Wave 3 of the PATH Study was 5.0% (95% CI: 4.7%–5.3%) (Table 1). Among all current ENP users (unweighted *n* = 2671), 43.9% used closed systems while 53.7% used open systems. Compared to closed system users, open system users were more likely to be male (60.7% vs. 48.4%), aged 18–24 (30.4% vs. 21.4%), and non-Hispanic White (76.2% vs. 65.4%) (Table 1). Over half of all current ENP users (58.9%) currently smoked cigarettes. Open system users were less likely than closed system users to be current daily smokers (34.3% vs. 48.0%) or report current use of other combusted products (26.2% vs. 37.2%), or never smokers (15.2% vs. 19.5%), and more likely to be recent former smokers (9.9% vs. 5.6%) or long-term former smokers (20.2% vs. 10.9%). Additionally, open system users were more likely than closed system users to endorse use of their device “because ENPs help people quit smoking cigarettes” (80.4% vs. 62.6%) or as a way of reducing cigarette smoking (79.1% vs. 59.7%).

Patterns of use and device modifications also varied by primary device type (Table 2). Open system users were more likely than closed system users to use ENPs daily (44.1% vs. 22.5%). Conversely, over three-quarters of closed system users (77.5%) were non-daily users of ENPs compared to 55.9% of open system users. Over half of current users reported past 30-day use of a fruit/sweet/spice/alcohol flavor. Open system users were more likely than closed system users to use a fruit/sweet/spice/alcohol flavor (67.8% vs. 34.8%) in the past 30 days, whereas closed system users were more likely to use tobacco flavor (37.4% vs. 15.7%) or menthol (25.7% vs. 15.0%).

## 4. Discussion

These data suggest that adult ENDS consumers at Wave 3 of the PATH Study (2015–2016) were nearly evenly split in their preferences for open vs. closed system device types (53.7% vs. 43.9%). In-depth examination of users suggests key differences between these groups in their characteristics and patterns of use. For instance, open system users were more likely to be male and aged 18–24. Consistent with prior research [5,7] those who used open system devices were also more likely to report higher frequency of use and use of fruit/sweet/spice/alcohol flavors vs. traditional flavors (i.e., tobacco or menthol), and less likely to be current cigarette smokers. Differences in cigarette smoking status may be understood, in part, by considering reasons for use: open system users were more likely than closed system users to endorse using ENDS “because it helps people quit smoking cigarettes”, or as a way to cut down on cigarette smoking.

In the earlier stages of ENDS product development, nicotine delivery was largely correlated with device type: namely, nicotine delivery was more effective in larger, more customizable (open system) devices compared to closed systems [12]. However, recent innovations in product design and nicotine delivery—exemplified by JUUL—reflect an evolving marketplace, providing consumers with options that offer the convenience and ease of use of a closed system combined with more effective nicotine delivery. Consistent with the current study findings demonstrating a near-even split between device types, qualitative research conducted in 2016 with adult ENDS users found that users of different device types (cigalikes [closed systems] and tanks [open systems]) have different priorities regarding device features (e.g., convenience vs. customizability, respectively)—which may relate, in part, to differing motivations for use [4]. As product innovation continues, research should continue to monitor if and how ENDS users of different devices vary in their patterns of use of ENDS and other tobacco products.

Given the cross-sectional nature of this analysis, we were unable to identify temporal relationships that could illuminate the potential causal effects of device type on smoking behavior (i.e., the timing of smoking cessation and established use of a particular device type). Moreover, other group differences observed (e.g., demographic, behavioral) could confound the relationship between ENDS use and smoking. Additionally, there are inherent challenges to categorizing device types and creating definitions using individual device attribute items in the Wave 3 PATH Study Adult Interview. To mitigate this problem, we identified a minimal set of key device features to distinguish device types based on current literature [4,11].

## 5. Conclusions

This study provides the first nationally-representative estimates on U.S. adult ENDS use by device type—i.e., open versus closed system users. In 2015–2016, this study found that current users were generally split in their preference for these device type categories. Of note, cigarette smoking status and reasons for use varied between users of different device types; open system users were more likely than closed system users to be former (vs. current) smokers and more likely to endorse using their device to help reduce cigarette smoking. Indeed, individuals vary in their motivations for using ENDS, and in turn, the devices they use may be more likely to promote certain behaviors (e.g., facilitate cigarette smoking). Disentangling the relationship between device selection and subsequent use patterns remains a priority, including identifying product characteristics that might be associated with increased likelihood of complete switching from cigarette smoking.

## Figures and Tables

**Table 1:** Demographic Characteristics of Adult Current Electronic Nicotine Product (ENP) Users by Device Type, 2015–2016.

	Overall (*n* = 2671)	Closed Systems ^a^ (*n* = 1153)	Open Systems ^b^ (*n* = 1446)	*p*-Value
	% (95% CI)	% (95% CI)	% (95% CI)	
Sex				**<0.0001**
Males	55.2 (52.9, 57.4)	48.4 (45.2, 51.7)	60.7 (57.9, 63.5)	
Females	44.8 (42.6, 47.1)	51.6 (48.3, 54.8)	39.3 (36.5, 42.1)	
Age group (years)				**<0.0001**
18–24	26.4 (24.6, 28.4)	21.4 (19.1, 24.0)	30.4 (27.2, 33.3)	
25–34	26.3 (24.3, 28.4)	26.5 (23.3, 29.9)	25.9 (23.2, 28.8)	
35–44	17.6 (15.9, 19.4)	15.7 (13.4, 18.4)	19.2 (16.9, 21.7)	
45–54	13.7 (12.2, 15.3)	15.0 (12.9, 17.4)	12.7 (10.8, 14.9)	
55–64	11 (9.7, 12.5)	14.4 (12.0, 17.2)	8.4 (7.0, 10.0)	
65+	5.0 (3.9, 6.5)	7.0 (5.1, 9.4)	3.4 (2.2, 5.3)	
Race/ethnicity				**<0.0001**
White, non-Hispanic	71.1 (68.8, 73.4)	65.4 (61.8, 68.8)	76.2 (73.4, 78.9)	
Black, non-Hispanic	9.4 (8.1, 10.8)	12.3 (10.3, 14.5)	6.5 (5.1, 8.3)	
Asian, non-Hispanic	2.9 (2.0, 4.2)	3.4 (2.0, 5.6)	2.6 (1.6, 4.2)	
Other, non-Hispanic	3.9 (3.2, 4.8)	3.6 (2.6, 4.8)	4.2 (3.3, 5.5)	
Hispanic	12.7 (11.4, 14.1)	15.4 (13.2, 17.8)	10.4 (8.9, 12.1)	
Education				
Less than high school diploma	12.4 (11.1, 14.0)	12.4 (10.4, 14.8)	12.3 (10.5, 14.5)	0.0978
GED	8.2 (7.1, 9.4)	9.3 (7.5, 11.4)	7.3 (5.8, 9.1)	
High school diploma	25.6 (23.5, 27.9)	26.2 (23.3, 29.3)	25.0 (22.1, 28.1)	
Some college/associate’s degree	40.3 (38.2, 42.4)	37.4 (34.2, 40.7)	43.0 (40.2, 45.7)	
Bachelor’s degree or more	13.4 (11.9, 15.0)	14.8 (12.3, 17.6)	12.5 (10.8, 14.4)	
Income ^c^				**0.0366**
<100% of the FPL	37.5 (34.8, 40.2)	40.6 (36.8, 44.5)	34.4 (31.1, 37.8)	
100%–199% of the FPL	26.1 (24.1, 28.3)	25.7 (22.3, 29.3)	26.7 (23.8, 29.7)	
≥200% of the FPL	36.4 (33.9, 39.0)	33.7 (30.3, 37.4)	39.0 (35.5, 42.5)	
Current use of other combusted products ^d^				**<0.0001**
Yes	31.6 (29.1, 34.2)	37.2 (33.9, 40.8)	26.5 (23.5, 29.9)	
Current use of non-combusted products ^e^				0.7856
Yes	8.8 (7.5, 10.4)	9.2 (7.0, 12.1)	8.6 (7.0, 10.4)	

*Note*. Boldface indicates statistical significance (*p* < 0.05) from Rao-Scott chi-square test. Frequencies reflect unweighted data and percentages reflect weighted data. Among the 2671 current (every day or some days) adult ENP users at Wave 3, 72 were dropped from the device type stratification due to either missing data on device attributes (*n* = 30) or incompatible combinations of responses that precluded device type categorization (*n* = 42). Of these 72 individuals who were dropped from the analysis, 43 (60%) were considered “experimental users”—i.e., have never used the product “fairly regularly.” Responses with “don’t know” and “refused” were treated as missing. Relatively few ENP users had missing data for variables in this study, usually less than 2%. ^†^ Estimate was flagged based on a Relative Standard Error (RSE) ≥ 30%. ^a^ Devices that are not rechargeable; or devices that are rechargeable and use cartridges. ^b^ Devices that are rechargeable, do not use cartridges, and are refillable. ^c^ Income information obtained at Wave 1. About 8% of ENP users did not have income information. ^d^ Respondents who have ever used (combustible product(s)), currently use it every day or some days. Combustible products include traditional cigars, cigarillos, filtered cigars, pipes, and hookah. ^e^ Respondents who have ever used (non-combustible product(s)), currently use it every day or some days. Non-combustible products include smokeless tobacco (snus pouches, loose snus, moist snuff, dip, spit, or chewing tobacco). CI, confidence interval; GED, General Education Development certificate; FPL, Federal Poverty Level for income.

**Table 2:** Patterns of Electronic Nicotine Product (ENP) Use among Adult Current Users (*n* = 2671) by Device Type, 2015–2016.

	Overall (*n* = 2671)	Closed Systems ^a^ (*n* = 1153)	Open Systems ^b^ (*n* = 1446)	*p*-Value
	% (95% CI)	% (95% CI)	% (95% CI)	
Prevalence	5.0 (4.7, 5.3)	2.2 (2.0, 2.4)	2.7 (2.6, 2.9)	
Overall	--	43.9 (41.3, 46.4)	53.7 (51.1, 56.2)	
Year(s) since first used ENP ^c^				0.9279
0	31.3 (27.8, 35.0)	29.7 (23.8, 36.4)	32 (27.8, 36.5)	
1	36.1 (32.6, 39.7)	36.1 (30.6, 42.0)	35.3 (31.8, 41.1)	
2–3	25.6 (22.2, 29.4)	26.6 (20.6, 33.7)	25.1 (21.3, 29.2)	
4–5	3.4 (2.3, 5.0)	3.2 (1.4, 6.9)	3.4 (2.2, 5.4) †	
6+	3.6 (2.2, 5.7)	4.4 (2.2, 8.7) †	3.2 (1.6, 6.1) †	
Frequency of use				**<0.0001**
Daily	34.0 (31.8, 36.3)	22.5 (19.9, 25.3)	44.1 (40.9, 47.3)	
Non-daily	66.0 (63.7, 68.2)	77.5 (74.7, 80.1)	55.9 (52.7, 59.1)	
Cigarette smoking status				**<0.0001**
Current daily smoker	40.5 (38.3, 42.7)	48.0 (44.5, 51.4)	34.3 (31.7, 37.3)	
Current non-daily smoker	18.4 (16.9, 20.1)	16.0 (13.7, 18.6)	20.3 (17.9, 23.0)	
Recent former (≤ 1 year)	8.0 (6.8, 9.3)	5.6 (4.3, 7.4)	9.9 (8.2, 11.9)	
Long-term former (>1 year)	15.8 (14.0, 17.8)	10.9 (9.1, 13.0)	20.2 (17.3, 23.3)	
Never smoker	17.3 (15.5, 19.3)	19.5 (16.5, 23.0)	15.2 (13.2, 17.4)	
ENP contains nicotine ^d^				**<0.0001**
Yes	78.4 (76.2, 80.4)	71.8 (68.2, 75.0)	84.7 (82.6, 86.6)	
Flavors used in the past 30 days				**<0.0001**
Tobacco flavor	24.7 (22.1, 27.5)	37.4 (33.2, 41.8)	15.7 (13.2, 18.6)	
Menthol or mint	19.4 (17.6, 21.2)	25.7 (22.3, 29.5)	15.0 (13.0, 17.2)	
Fruit/sweet/spice/alcohol flavor ^e^	54.0 (51.2, 56.8)	34.8 (30.8, 39.1)	67.8 (64.6, 70.8)	
Some other flavor (specify)	1.9 (1.4, 2.7)	2.1 (1.3, 3.4)	1.5 (0.9, 2.4)	
Ability to change voltage of their device ^f^				**<0.0001**
Yes	53.0 (50.1, 55.8)	24.8 (21.2, 28.8)	68.1 (64.9, 71.1)	
No	35.8 (32.9, 38.6)	56.3 (51.2, 61.3)	25.3 (22.6, 28.3)	
Don’t know	11.2 (9.7, 13.0)	18.9 (15.2, 23.3)	6.6 (5.3, 8.3)	
Changes voltage of their device ^g^				**0.0226**
Yes	71.7 (68.3, 75.0)	61.6 (52.5, 69.9)	73.5 (69.7, 77.0)	
No	24.8 (21.6, 28.3)	33 (25.4, 41.7)	23.4 (20.1, 27.2)	
Don’t know	3.4 (2.1, 5.5)	5.4 (2.9, 9.9) †	3.0 (1.7, 5.4) †	
Endorsed using ENDs because “[ENDS] helps people quit smoking cigarettes” ^h^				**<0.0001**
Yes	71.8 (69.7, 73.9)	62.6 (58.3, 66.8)	80.4 (77.3, 83.2)	
Uses [ENDS] as a way of cutting down on cigarette smoking ^i^				**<0.0001**
Yes	69.5 (66.9, 72.0)	59.7 (55.8, 63.5)	79.1 (76.1, 81.8)	
Time to first [ENDS] within 30 min of waking				0.1438
Yes	43 (40.6, 45.6)	44.4 (40.9, 48.0)	41.0 (37.9, 44.3)	
Considers themselves addicted to [ENDS] ^j^				**0.0001**
No, not at all	63.2 (60.4, 66.0)	70.5 (66.1, 74.6)	58.6 (55.1, 62.1)	
Yes, somewhat addicted	29.9 (27.3, 32.6)	22.1 (18.9, 25.7)	34.7 (31.3, 38.3)	
Yes, very addicted	6.9 (5.4, 8.7)	7.4 (4.5, 12.0)	6.7 (5.3, 8.3)	
Quit attempt of ENP in the past year ^k^				**<0.0001**
Yes	12.8 (11.1, 14.7)	16.3 (13.3, 19.8)	10.5 (8.7, 12.6)	

*Note*. Boldface indicates statistical significance (*p* < 0.05) from Rao-Scott chi-square test. Frequencies reflect unweighted data and percentages reflect weighted data. Among the 2671 current (every day or some days) adult ENP users at Wave 3, 72 were dropped from the device type stratification due to either missing data on device attributes (*n* = 30) or incompatible combinations of responses that precluded device type categorization (*n* = 42). Of these 72 individuals who were dropped from the analysis, 43 (60%) were considered “experimental users”—i.e., have never used the product “fairly regularly.” Responses with “don’t know” and “refused” were treated as missing. Relatively few ENP users had missing data for variables in this study, usually less than 2%. ^†^ Estimate was flagged based on a Relative Standard Error (RSE) ≥ 30%. ^a^ Devices that are not rechargeable; or devices that are rechargeable and use cartridges. ^b^ Devices that are rechargeable, do not use cartridges, and are refillable. ^c^ Year(s) since first used ENP were calculated by subtracting the age first used ENP fairly regularly from current age. Age first used fairly regularly was obtained in either Wave 1, Wave 2 or Wave 3. These questions were asked to those who are current established users *n* = 794). ^d^ Used question, “Does/Did the ENP product you use/used contain nicotine?” (*n* = 2642). ^e^ Fruit/sweet/spice/alcohol flavors include clove or spice, fruit, chocolate, “an alcoholic drink (such as wine, cognac, margarita or other beverages, candy, desserts, or other sweets).” (*n* = 2193). ^f^ Respondents who responded “yes” to having the ability to change the voltage on their device were asked the following question: “Can/Could you change the voltage on your product?” (*n* = 2183). ^g^ Only included respondents who reported the ability to change the voltage on their device (*n* = 1165). ^h^ Respondents were asked, “Using ENDS helps people quit smoking cigarettes” (*n* = 1900). ^I^ Respondents were asked, “Do/Did you use ENDS as a way of cutting down on your cigarette smoking?” (*n* = 1715). ^j^ Respondents were asked, “Do/Did you consider yourself addicted to electronic nicotine product?” (*n* = 1781). For footnote h, i, j, a skip pattern error for variable R03_EPROD_USER_STATUS (adult e-product use status) resulted in some respondents incorrectly being skipped out of questions about reasons for use. See the Population Assessment of Tobacco and Health (PATH) Study [United States] Restricted-Use Files Wave 3: Adult Questionnaire Data (https://www.icpsr.umich.edu/icpsrweb/NAHDAP/studies/36231/datadocumentation#) for more details. ^k^ Quit attempt was defined using question, “In the past 12 months, have you tried to quit [e-product] completely?” (*n* = 1720) Skip pattern error for variable R03_EPROD_USER_STATUS (adult e-product use status) resulted in some respondents incorrectly being skipped out of questions about quit attempt. See the Population Assessment of Tobacco and Health (PATH) Study [United States] Restricted-Use Files Wave 3: Adult Questionnaire Data (https://www.icpsr.umich.edu/icpsrweb/NAHDAP/studies/36231/datadocumentation#) for more details.
